# Cross-modality sub-image retrieval using contrastive multimodal image representations

**DOI:** 10.1038/s41598-024-68800-1

**Published:** 2024-08-13

**Authors:** Eva Breznik, Elisabeth Wetzer, Joakim Lindblad, Nataša Sladoje

**Affiliations:** 1 Department of Information Technology, Uppsala University, 751 05 Uppsala, Sweden; 2 Department of Biomedical Engineering and Health Systems, Royal Institute of Technology, 141 52 Stockholm, Sweden; 3 Department of Physics and Technology, UiT The Arctic University of Norway, 9037 Tromsø, Norway

**Keywords:** Computational biology and bioinformatics, Image processing, Machine learning

## Abstract

In tissue characterization and cancer diagnostics, multimodal imaging has emerged as a powerful technique. Thanks to computational advances, large datasets can be exploited to discover patterns in pathologies and improve diagnosis. However, this requires efficient and scalable image retrieval methods. Cross-modality image retrieval is particularly challenging, since images of similar (or even the same) content captured by different modalities might share few common structures. We propose a new application-independent content-based image retrieval (CBIR) system for reverse (sub-)image search across modalities, which combines deep learning to generate representations (embedding the different modalities in a common space) with robust feature extraction and bag-of-words models for efficient and reliable retrieval. We illustrate its advantages through a replacement study, exploring a number of feature extractors and learned representations, as well as through comparison to recent (cross-modality) CBIR methods. For the task of (sub-)image retrieval on a (publicly available) dataset of brightfield and second harmonic generation microscopy images, the results show that our approach is superior to all tested alternatives. We discuss the shortcomings of the compared methods and observe the importance of equivariance and invariance properties of the learned representations and feature extractors in the CBIR pipeline. Code is available at: https://github.com/MIDA-group/CrossModal_ImgRetrieval.

## Introduction

Content-based image retrieval (CBIR) systems are designed to search images in large databases based on *content*. Queries may be provided in various forms such as class labels, key words and images. The type of CBIR using images as queries is termed Reverse Image Search (RIS), also known as *query-by-example*. CBIR systems traditionally consist of a feature extraction method followed by matching based on a suitable similarity measure^1,2^. Following the advent of deep learning, feature extraction is often performed by convolutional neural networks (CNNs), sometimes using pretrained networks^3–6^. When only a small patch of an image is provided as a query, the CBIR is termed sub-image retrieval (s-CBIR).

Often local feature descriptors are accumulated into a bag-of-words (BoW)^7–9^, where the most descriptive features (words) form a vocabulary and each image is assigned a histogram of words. The retrieval step is then based on histogram comparison, typically using cosine similarity. In some approaches, global features are used in the first stage of image retrieval to find the most similar images within a dataset whereafter the top results are re-ranked using local features^10^.

CBIR systems have gained popularity in digital pathology^11–13^ due to the increased use of whole slide image (WSI) scanners which enable lowered storage costs of glass slides, simplified transportation of samples, training of new experts, spatial navigation of the sample^14^, and powerful computer-assisted sample analysis^15^. By supporting efficient searches through the huge datasets, CBIR techniques facilitate diagnostic decision-making through easy access to similar cases and potentially unravel patterns useful for early diagnosis of diseases such as cancer^15^.

WSI scanners generally capture a single modality, usually fluorescent or brightfield (BF) microscopy. Acquiring additional images by different sensors may provide highly relevant complementary information. However, with the explicit aim to capture *different* types of information, the acquired images may have very different appearances and share few structures. For many medical diagnoses, in particular cancer diagnosis and grading, manual examination of tissue samples with a hematoxylin and eosin (H&E) stain using BF microscopy^16^ is the gold standard. In recent years, the label-free, non-linear imaging modality of second harmonic generation (SHG) proved to be useful for diagnostics for a variety of tissues, such as skin, ovaries and breast among others^17^. To facilitate content understanding, SHG images are often inspected side by side with corresponding BF images. To fully exploit the advantages of such (large) multimodal datasets, the ability to query them across modalities can serve as a very important and useful tool to aid the diagnostic process. Furthermore, WSI scanners capture a large tissue area, often up to $100,000 \!\times \! 100,000$ px, while SHG imaging at the same scale can typically only cover smaller areas. Hence SHG images can be taken at various, chosen locations within the tissue sample and provide local samples of additional information to a WSI BF image. To match the locations of the complimentary imaging within the WSI, cross-modal sub-image retrieval methods can return the most likely sites of acquisition – thereby accelerating a very time-consuming task if it were done fully manually, taking a step towards automated imaging and multimodal image fusion.

**Figure 1:**
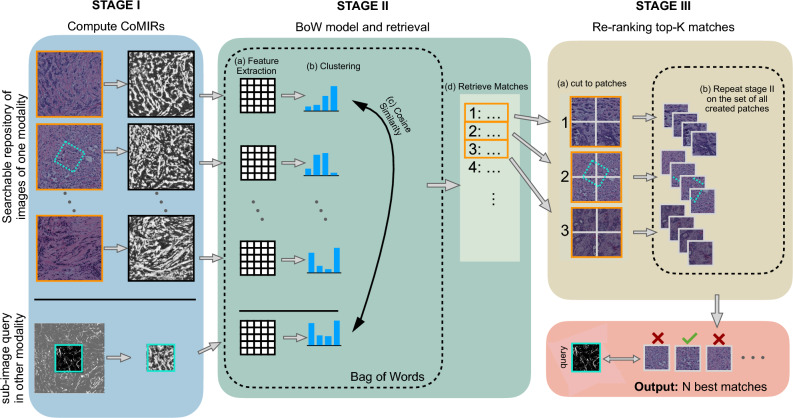
Illustration of the proposed three-stage s-CBIR pipeline. Stage I includes learning CoMIRs for the images in the repository and the query (either as a fullsized image or a patch), followed by sparse feature extraction in Stage II, which are binned into single descriptors for each image, building the vocabulary for a BoW. Matches are found using the cosine similarity. In Stage III, the Top-K matches are split into patches and a new BoW is computed on them for Re-Ranking.

Cross-modality image retrieval (CMIR), i.e. retrieval of images in one modality or visual domain provided a query in another, is also referred to as *cross-domain image retrieval* (CDIR) or *cross-source image retrieval*. A recent review on CDIR^18^ addresses work done in the field of near infrared & visible light retrieval used in person re-identification^19,20^, synthetic aperture radar & optical image as well as multispectral images & panchromatic images retrieval in remote sensing^21–23^, and sketch & natural image retrieval^24–28^. The methods used in these applications can be categorized into two types based on their approach to solving the domain gap problem^18^: feature space migration^3,4,6,19,21,23^ and image domain migration^22,25–28^. The first type extracts features of images in both modalities and attempts to find a mapping function (often through contrastive losses) to ensure feature similarity of corresponding image pairs or classes. The second relies on generative networks to translate one modality into the other^22,25,26,28^. However, these methods often employ domain-specific steps (e.g. incorporation of edge information in sketch retrieval), or focus on category-level retrieval (assuming a query corresponds to more than one image in the repository, i.e. to all images of the same category) relying on class labels with multiple samples per class. Hence they are not applicable to very different domains such as microscopy images or instance-level retrieval (where each query has a single correct match in the repository) in general. Hashing-based methods, which have been successfully used for image retrieval within medical imaging^29,30^, have also been applied to cross-modality retrieval^4,29^, but they generally use class labels for training, assuming multiple instances per class.

In this paper, we study cross-modality image retrieval formulated as RIS on an instance level, i.e. the images are unlabelled and the aim is to retrieve the image of modality *A* corresponding to a given query image of modality *B*, where images of different modalities have a one-to-one correspondence. This may occur if one modality cannot be acquired for the entire sample due to long imaging acquistion times but is imaged at random to supply additional information about the sample. Furthermore, such matching challenges also arise in satellite imagery where the same location is imaged at different times, with different modalities and fields of view. We propose a data-independent three-stage s-CBIR system that uses representation learning to transform images of both modalities into a common space via contrastive multimodal image representations for registration (CoMIRs)^31^, followed by feature extraction and a BoW model to perform retrieval in this abstract representation space. Finally, we suggest re-ranking the top results to further improve the performance.

The proposed approach is evaluated on the very challenging task of (sub-)image retrieval across the BF and SHG modalities, since these two modalities are too different in their appearances to enable successful retrieval using existing monomodal RIS approaches.

While our method was developed for this particular application, we also evaluate it on a separate dataset of aerial images. First, we perform a replacement study with several viable alternatives for each stage of the proposed method to illustrate the advantages of our particular pipeline design. In addition, we evaluate our method against recent state-of-the-art methods in cross-modality and biomedical image retrieval.

*Contributions*: We propose a state-of-the-art cross-modality sub-image retrieval system for RIS, combining CoMIR representation learning and SURF feature extraction. We carry out a replacement study to demonstrate its efficacy. Our proposed approach outperforms state-of-the-art methods on the challenging task of image retrieval across BF an SHG modalities. Furthermore, we: (i) discuss the shortcomings of the I2I based approaches and highlight the necessity of rotationally equivariant representations for translating the multimodal task into a monomodal one; (ii) demonstrate the importance of rotational invariance of the feature extractor; and (iii) show that re-ranking can boost the retrieval performance significantly. We share the code as open source at https://github.com/MIDA-group/CrossModal_ImgRetrieval.

## Methods

Our aim is to match corresponding areas of two different modalities which may be hard to align even by human inspection^31^. The proposed pipeline is modular, with three main stages. The first stage uses representation learning to bridge between the modalities, the second stage consists of feature extraction, bag of words computation and match retrieval, followed by re-ranking in the third stage, in which a new BoW of the top retrieval results is computed, as depicted in Fig. 1.

### Stage I: representation learning

We use representation learning to bridge the gap between the input modalities inspired by the success of other recent multimodal image retrieval approaches^22,25–28^. Our proposed pipeline does so by using contrastive learning (stage I in Fig. 1). Contrastive losses are used in a number of multimodal image retrieval tasks to learn feature embeddings which are similar for corresponding samples^21,27^. In the proposed method we use *contrastive multimodal image representations (called CoMIRs)*, which are image representations learned by training two CNNs in parallel with aligned image pairs of different modalities. Using a contrastive loss, the two networks produce representations of the input images, such that two CoMIRs resulting from corresponding areas in the two input modalities have maximum similarity w.r.t. a selected similarity measure. The networks are provided with randomly chosen $\{0^{\circ },90^{\circ },180^{\circ }, 270^{\circ }\}$-rotated versions of the input images, which are aligned with the corresponding input of the other modality in the second network before the contrastive loss is computed, thereby enforcing rotationally equivariant properties of the representations. The representations preserve common structures, which makes them useful for multimodal image registration and hence suitable candidates for image retrieval. For more details on the method and implementation see Appendix A.1 , and the original paper, Pielawski & Wetzer et al.^31^.

### Stage II: feature extraction and creation of BoW

This stage consists of extracting features from the CoMIR images and building a BoW model based on them. We employ Speeded Up Robust Features (SURF)^32^ (step (IIa) in Fig. 1) which are sparse, scale- and rotation invariant, hence they are expected to perform well even with rigidly transformed or cropped queries. The BoW is defined on the features extracted from the CoMIRs of all the images in the searchable repository, by K-means clustering using a suitable vocabulary (step (IIb) in Fig. 1). The features extracted from the CoMIR of the (rigidly transformed) query image are encoded using the created vocabulary, resulting in a histogram of features associated to the BoW. This histogram is then matched against the database using cosine similarity (steps (2c &2d) in Fig. 1) to retrieve the best matches.

### Stage III: re-ranking

To further improve the retrieval of our (s-)CBIR system, the best-ranked matches can be re-ranked. To do so, we take a predefined number of top retrieval matches (which are full images) and cut them into non-overlapping patches of the same size as the query. The resulting patches form a new database for which a new BoW model and (s-)CBIR ranking is computed, using the same configuration as the initial one (step (IIIb) in Fig. 1). For full-image search, no cutting into patches is needed. Instead, the new retrieval ranking is computed directly on the image subset consisting of the (predefined number of) top-ranked matches. Re-ranking can be done in multiple ways, however a recent smaller-scale study^33^ supports our re-ranking strategy choice.

## Evaluation

The aim is to retrieve a (transformed) query (sub-)image from a repository storing the other modality. A successful match is defined as the retrieval of the image corresponding to the query of the other modality (instance-level retrieval). To thoroughly evaluate the proposed method we run a replacement study on its individual modules, as well as compare it against current state-of-the-art in CMIR. The evaluation is performed for both full-sized and small patch queries and done exhaustively, using all images in the (one-modality) dataset as queries. Experiments are performed on two different datasets of microscopy (presented here) and aerial images (available in the Appendix A.2).

### Dataset

**Figure 2:**
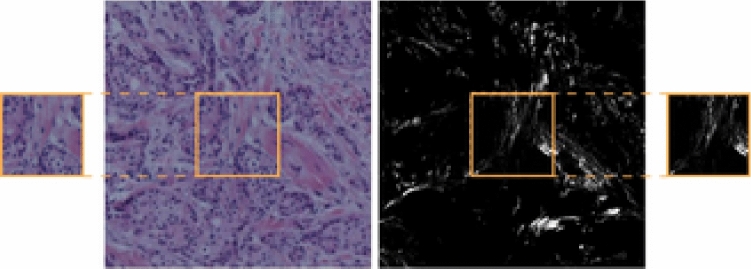
Example image pair used in the CBIR experiments (BF on the left, SHG on the right). This image pair is shown aligned, without the rotations and translations used in the test set. In addition, the smaller image cutouts show patches used in the s-CBIR experiments, with the orange squares indicating how the patches were cropped.

Our evaluation dataset consists of 206 BF and SHG image pairs of size $834 \times 834$ px, and is an openly available registration benchmark^34^ based on data collected at the University of Wisconsin-Madison and produced by Conklin et al.^35^ after approval from the institutional review board. For each image pair, also its rigidly transformed version is provided. The transformations consist of a random rotation up to $\pm 30^{\circ }$, and random translations up to $\pm 100$ px in *x* and *y*. Following Pielawski & Wetzer et al.^31^, 40 untransformed pairs are used for training, 134 for testing, and the remaining 32 for validation. SHG images were preprocessed by a log-transform for the I2I and CoMIR generation. For evaluating the s-CBIR, patches of size $256\times 256$ px are cropped from the centres of the $834\times 834$ px images. An example pair is shown in Fig. 2.

### Evaluation metric

As the evaluation metrics we use the *top-k retrieval success*, indicating for what fraction of queries a correct image was found in the first k matches (often denoted by Acc@k, accuracy at k). Formally, it can be defined as

1 $$\begin{aligned} \text{ Acc@k } = \frac{1}{N}\sum _{i=1}^N I_{i,k} \end{aligned}$$

where $I_{i,k}$ is an indicator function of whether the correct match for query *i* was present among the first *k* retrieved images. The other commonly used metrics within retrieval such as precision@k and (m)AP@k ((mean) average precision at k) are less appropriate due to the one-to-one correspondence of images. (The precision@k for example degenerated a binary measure of either 0 of 1/*k*). For $k=1$ however, all three metrics coincide in this case. Our choice of metric can be additionally motivated by the clinical background of our primary application (matching SHG and BF data). While finding the correct match as the first retrieved image is of course desirable, the most important and realistic contribution of an image retrieval method in such applications is in form a support tool; reliably identifying the correct match within a manageably small number of retrievals, enabling (lowering the load of) human/visual inspection.

### Experimental settings for the proposed method

To learn 1-channel CoMIRs, two U-Nets^36^ sharing no weights are used under the same settings as in Pielawski & Wetzer et al.^31^ for registration, with mean squared error as a critic, manually tuned temperature $\tau =0.5$ and 46 negative pairs in each iteration. The networks are trained on patches of size $128 \times 128$ px randomly extracted from 40 aligned training pairs. For more details on CoMIR training and experimental details see Appendix, A.1. The SURF features are extracted for patch sizes 32, 64, 96,  and 128 on a grid with spacing (8, 8), using a descriptor size of 64. The BoW is then defined on the features of the entire set of untransformed CoMIRs, using a large vocabulary of 20,000 words (based on empirical testing) on the $80\%$ of the strongest features and the matching is done with cosine similarity. We do the final re-ranking among top-15 and top-30 matches. For s-CBIR, the image is cut into the minimal number of equidistantly placed query-sized patches s.t. the image is fully covered.

### Replacement study

To confirm the design choices of our proposed pipeline, a replacement study is performed on stages I and II of the pipeline. Re-ranking is performed at the end, only where base results suggest its applicability (i.e. only within the proposed pipeline).

#### Stage I alternatives

For bridging the gap between modalities, CoMIR representations are compared against two generative adversarial network (GAN)-based image-to-image translation (I2I)^37,38^ methods, used to translate BF images into SHG images, and vice versa.

GANs consist of a generator and a discriminator, competing in a zero-sum game. The generator learns to generate a representation in one modality given the input in the other modality; the discriminator classifies the representation as generated or real, thereby training the generator to produce representations indistinguishable from real images. The resulting so-called “fake” BF and SHG images can be used as queries, enabling search in near-monomodality.

Pix2pix^37^ uses a conditional GAN to learn a mapping from an input image to a representation using corresponding images of both modalities, i.e. requires supervision in form of aligned multimodal image pairs. CycleGAN^38^ differs from pix2pix by achieving this goal even in an unsupervised manner, through a cycle consistency enforcing that the input image can be reconstructed from the representation that was produced based on it. CycleGAN-based domain adaptation has been used successfully in previous multimodal image retrieval tasks^22,28^. For both approaches, training parameters are used as in Pielawski & Wetzer et al. ^31^. The code is available at https://github.com/junyanz/pytorch-CycleGAN-and-pix2pix.

#### Stage II alternatives

For the second stage we compare our choice of SURF against two other commonly used feature extractors, SIFT^39^ and ResNet^40^. Regardless of the feature extractor choice, the BoW is defined on the features of the entire set of untransformed input images of one modality (or their learned representations such as CoMIRs), using a vocabulary of 20,000 words on the $80\%$ of the strongest features and the matching is done with cosine similarity. In addition, we test replacing the entire stage II (feature extraction and BoW) with a recent RIS s-CBIR toolkit 2DKD^41^, based on 2D Krawtchouk descriptors.

SIFT (Scale Invariant Feature Transform)^39^ is a well known feature extractor with similar properties as SURF, being sparse, scale and rotation invariant. In our evaluation the feature descriptor size is 4 samples per row and column, 8 bins per local histogram. The range of the scale octaves is [32, 512] px, with 4 steps per scale octave and an initial $\sigma$ of each scale octave equal to 1.6. The descriptor size is 128. We explore ResNet^40^ as a dense feature extractor based on previous successes of using features extracted by neural networks for image retrieval^13,42–44^. As data used in this study is not required to have labels, we extract features by ResNet152, pretrained on ImageNet^44^ (see https://pytorch.org/vision/stable/models.html). The features are extracted by removing the last fully connected layer. To enable patch queries, an adaptive average pooling is added to produce features of size $8\times 8$ (64 when flattened), independent of the input size. The number of extracted features is 2048 regardless of the input image size. For SHG, the image is copied into three channels. As opposed to using SIFT/SURF which extract an arbitrary number of features from every image, the amount of ResNet extracted features is the same for every image (or patch). 2DKD Toolkit^41^ is a recent RIS system which differs from the BoW approach in that it is performing a local sub-image search using a number of translation, rotation, and scaling invariant descriptors per image. It relies on moment invariants based on Krawtchouk polynomials^45^, namely Two Dimensional Krawtchouk Descriptors, which outperform Hu invariants in retrieving subimages in cryo-electron microscopy images in a monomodal setting^41^. The authors point out the importance of using moment invariants that do not change by translation, rotation and scaling in digital pathology. In our evaluation, the number of pixels between two consecutive points of interest is set to 5 and local pixel intensity variance is used as a criterion to compare against global pixel density variance. The general experimental setup follows the one of DeVille et al.^41^.

### Competing methods

Since methods for instance-level cross-modality retrieval (specifically applicable in the biomedical field) are scarce, we compare our method to (1) a recent CMIR method, Triplet Classification Network^24^ (TC-Net) which was developed primarily for retrieval across sketches and natural images but is not domain-specific in design and can be used for instance-level retrieval, and (2) a medical image retrieval system based on invariant moments, textural and deep features ^5^ (IMTDF), which has been developed for, and evaluated on, within-modality retrieval, but relies in part on a study suggesting cross-modal applicability^4^. While more recent CMIR methods exist, they are not applicable for our particular evaluation task.

TC-Net improves on the previous works^46^ in cross-modality sketch retrieval by circumventing the generation of edge maps, as their quality has a large effect on CBIR system performance. It uses a triplet Siamese network, and auxiliary classification losses. For the evaluation, we use the settings of the original paper^24^, however for a fairer comparison we train the network with BF anchors for retrieval within SHG database, and with SHG anchors for retrieval within BF database.

IMTDF relies on a combination of various invariant moments, classical texture features and CNN based features. It thereby follows other RIS methods in biomedical applications which rely on CNN features^3,4^ for both mono- and cross-modal retrieval, and their combination with classical features, as successfully used for cross-modal retrieval of magnetic resonance imaging (MRI) and computed tomography (CT) images^5^. Based on the best performing features reported for IMTDF^5^, we use a combination of Chebyshev moments, Haralick texture features (made rotationally invariant), and ResNet50 features, selecting only the strongest $20\%$ of the latter two by means of ReliefF. For more details and parameter settings see the original paper^5^.

**Table 1:**
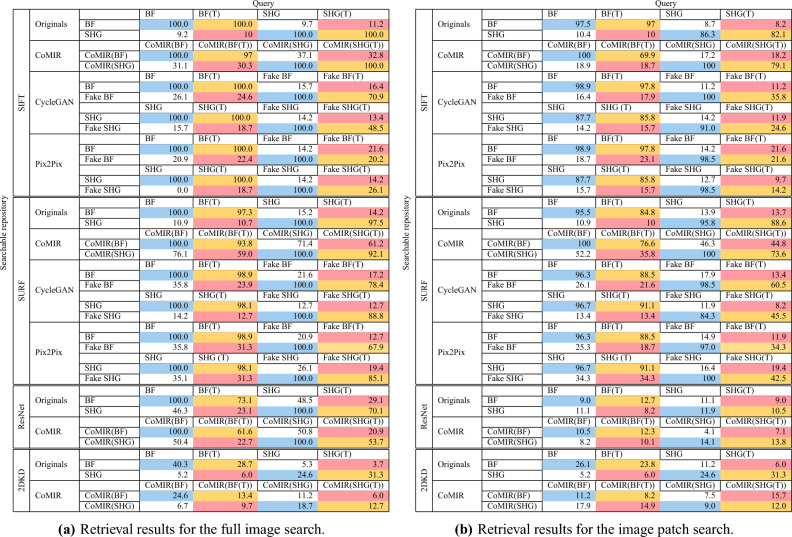
Main results of the replacement study: success (in percentage) for top-10 match.

BF and SHG represent the two original modalities, *fake SHG* and *fake BF* represent their corresponding I2I representations produced by CycleGAN or pix2pix, and (T) denotes randomly transformed (by rotation and translation) images. Best results for retrieval across modalities and transformations are marked in bold.

## Results

### Replacement study

Table 1a shows the results of the proposed CBIR using full sized query images. It reports the top-10 retrieval success in percentage using the multimodal originals, CoMIRs, and I2I representations produced by CycleGAN and pix2pix as searchable repositories and queries. Experiments were performed using SIFT, SURF and ResNet features to create the vocabulary of the BoW. Performance of 2DKD is evaluated on the multimodal original images as well as CoMIRs. For evaluation of sub-image retrieval in the s-CBIR setup, the same experiments are performed using central patches of $256 \times 256$ px as query images. Results are shown in Table 1b. Retrieval results of I2I representations in combination with ResNet features and using 2DKD are omitted from the table, as they resulted in fewer retrieval matches than random selection.

**Figure 3:**
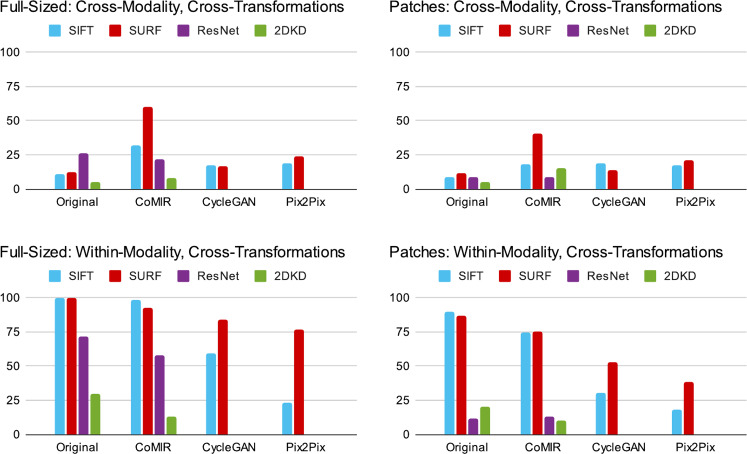
Top-10 retrieval results without re-ranking, for full-sized image queries (left column) and patches (right column), averaged over retrieval directions (BF query within SHG and SHG query within BF, or their respective representations) for different combinations of images or their learned representations and feature extractors. Cross-modality (top row) and within-modality (bottom row).

In Table 1, red cells present the results of cross-modality retrieval of a transformed (rotated and translated) image in one modality among a set of untransformed images *of the other modality*, which is the main use-case targeted by this study (denoted cross-modality, cross-transformations subsequently in Fig. 3 and Table 2a). Orange cells present the results of retrieving a transformed query image within the *same modality* of untransformed images. This gives insight into the invariance of the feature extraction, or the equivariance of the representations under these transformations (denoted within-modality, cross-transformations subsequently in Fig. 3 and Table 2a). Blue cells present the results of searching an untransformed query image within the *same modality* of untransformed images, which validates the CBIR setup. A near-perfect retrieval accuracy indicates that the features extracted to create the BoW and its vocabulary size are reasonable. White cells present the results of retrieving an untransformed query image among images of *the other modality*, which shows how well the learned representations are bridging the semantic gap between the modalities.

Figure 3 summarizes the results for full-sized and patch queries, with numbers from Table 1a, b averaged over retrieval directions (BF query within SHG and SHG query within BF).

*Re-ranking* Re-ranking is evaluated only as a part of the proposed pipeline: with using SURF on CoMIRs to create a BoW. In Table 2a, we see that re-ranking among the top-15 retrieval matches can boost the top-10 retrieval performance for full-sized images, increasing retrieval accuracy to 62.0% (BF as query) and 66.4% (SHG as query); or 75.4% and 83.6% respectively for re-ranking among the top-30 matches. Table 2b shows that re-ranking among the top-15 retrieval matches can boost the top-10 retrieval performance also for patch queries, increasing retrieval accuracy to 41.8% (BF as query) and 53.7% (SHG as query); or to 51.5% and 63.4% respectively for re-ranking among the top-30 matches.

**Table 2:** Performance gain for full size (left) and patch queries (right) due to re-ranking among the top-15 and top-30 matches of the main pipeline using SURF features on CoMIRs.

Full sized	Cross-modality, cross-transformations				
		Top-1	Top-5	Top-10	Top-15
(a) Full size queries					
CoMIR(SHG)	query: CoMIR(BF(T))				
	No re-ranking	26.9	51.5	59.0	63.4
	Re-Ranking 15	32.8	55.2	62.0	63.4
	Re-Ranking 30	34.3	59.7	75.4	75.4
CoMIR(BF)	query: CoMIR(SHG(T))				
	No re-ranking	21.6	45.5	61.2	69.4
	Re-Ranking 15	42.5	57.5	66.4	69.4
	Re-Ranking 30	45.5	64.9	83.6	83.6

Patches	Cross-modality, cross-transformations				
		Top-1	Top-5	Top-10	Top-15
(b) Patch queries					
CoMIR(SHG)	query: CoMIR(BF(T))				
	No re-ranking	11.2	25.4	35.8	42.5
	Re-Ranking 15	23.1	37.3	41.8	42.5
	Re-Ranking 30	27.6	47.0	51.5	55.9
CoMIR(BF)	query: CoMIR(SHG(T))				
	No re-ranking	10.4	32.8	44.8	53.7
	Re-ranking 15	29.1	46.3	53.7	53.7
	Re-ranking 30	29.1	53.7	63.4	67.2

### Comparison with state-of-the-art

Among the latest state-of-the-art methods in CMIR, few are applicable to instance-level retrieval between modalities as different as the ones used in our evaluation dataset. IMTDF relies not only on ResNet features, but also on a number of handcrafted features that perform well for within-modality retrieval on medical images. As seen in Table 3 however, this combination of features does not perform well for cross-modality retrieval of BF and SHG images. TC-Net on the other hand has been previously evaluated on modalities very different from BF and SHG, but is generally not domain specific. It is based on a similar training mechanism as the representation learning stage in our proposed method and outperforms IMTDF on retrieval across BF and SHG (see Table 3). However, our proposed method outperforms both TC-Net and IMTDF, even without re-ranking.

### Generalization to other datasets

To show that the proposed pipeline is modality-agnostic and not taking any biological knowledge into consideration, additional experiments run on aerial images are available in the Appendix A.2. Our proposed method reaches 91.9% average top-1 retrieval accuracy, outperforming both competing methods and pipeline design variations.

**Table 3:** Top-10 retrieval success (in percentage) of two state-of-the-art RIS methods on (sub-)image retrieval across BF &SHG modalities.

		Query			
		BF	BF(T)	SHG	SHG(T)
(a) Full size queries					
	Searchable repository				
Proposed	BF	–	–	89.6	83.6
	SHG	86.6	75.4	–	–
TC–Net	BF	–	–	47.8	43.4
	SHG	35.8	37.3	–	–
IMTDF	BF	–	–	9.7	11.9
	SHG	12.7	11.2	–	–
(b) Patch queries					
	Searchable repository				
Proposed	BF	–	–	66.4	63.4
	SHG	56.7	51.5	–	–
TC-Net	BF	–	–	22.4	20.1
	SHG	12.7	16.4	–	–
IMTDF	BF	–	–	7.5	7.5
	SHG	7.5	8.2	–	–

Here reported retrieval success of our proposed pipeline includes top-30 reranking.

## Discussion

Our study demonstrates that out of all tested settings and methods, our proposed pipeline is the best-performing one for the task at hand, yielding a 61.2% top-10 success rate retrieving BF queries in a set of SHG images and 59.0% retrieving SHG queries within the set of BF images. With re-ranking the first 30 matches, these results are further improved to 75.4% and 83.6% respectively. The strength of combining learning based CoMIRs with classic feature extractors such as SURF, merges the potential of CNNs to produce equivariant representations which can bridge between different modalities, and robust, sparse feature extractors that are rotationally and translationally invariant, and due to their speed qualify for creating (s-)CBIR systems for large datasets. Moreover, CoMIR and SURF are modality-independent, not incorporating any data-specific information, which makes the pipeline generally applicable, which is demonstrated by experiments on an aerial dataset in Appendix A.2.

**Figure 4:**
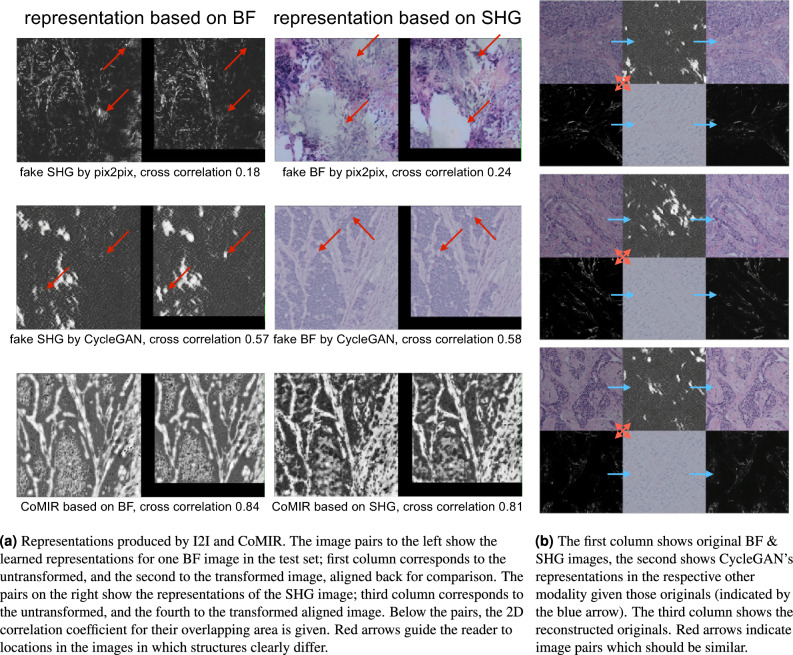
Visual examples of the test set demonstrating the shortcomings of the I2I approaches to bridge the semantic gap between the modalities. **a** Translational and rotational equivariance is not preserved for I2I generated images, **b** the fake BF images (even rows, middle) do not preserve the structure and appearance of the corresponding real BF images, but appear similar, independent of the content of the SHG images they are generated from.

As seen in Table 1a, performing within-modality full image retrieval on the original images with transformed queries has a 100% or close to 100% success rate when using the SURF or SIFT feature extractors, while using ResNet as a feature extractor results in a significant drop in retrieval success. This can be attributed to the lack of rotational invariance of ResNet as a feature extractor. While this high within-modality retrieval success is retained when using CoMIR embeddings (i.e. retrieving the CoMIR of a transformed image in modality *A* within the set of CoMIRs of untransformed images of modality *A* and vice versa), it drops significantly with the use of I2I approaches (i.e. retrieving the fake GAN image of a transformed image in modality *A* within the set of CoMIRs of untransformed images of modality *A* and vice versa) even when using SURF or SIFT features. Since SIFT and SURF are rotationally invariant by design, we argue that the reduced performance when using them in combination with I2I approaches is due to the GAN-generated images not preserving translational, and in particular rotational, equivariances in their representations. The reason behind this shortcoming of the GAN-generated images is the absence of network architecture related enforcement (as there is with, e.g., steerable CNNs or group convolutions) in place for pix2pix or CycleGAN to relate rotated versions of the input with each other. As long as their generated fake images belong to the distribution of the target modality, the discriminator will accept them as reasonable output. Figure 4a shows an example from the test set illustrating this effect. As seen by the cross-correlation of their (aligned) overlap, the fake representations of the untransformed and transformed images can differ significantly.

On the other hand, attempting cross-modality retrieval directly on the original images fails regardless of the choice of feature extractor, thus highlighting the need for bringing the two modalities closer together. While bridging the gap between SHG and BF modalities through CoMIRs delivers strongly improved cross-modality retrieval success when using SIFT or SURF, the I2I approaches are less advantageous. We notice that CycleGAN suffers from so-called mode-collapse. In Fig. 4b, three examples are shown for which the fake BF modalities (middle image in row 2,4 and 6) are extremely similar, independent of the input images. The structures in the original BF images are not preserved, instead texture was generated that is accepted by the discriminator as a reasonable BF tissue. While these fake BF images successfully encode the information required to reconstruct the SHG images, fulfilling the cycle consistency (third column in Fig. 4b), they failed to produce a representation similar to the real target BF image.

The rotationally equivariant CoMIRs together with invariant feature extractors like SIFT and SURF can handle the displacements between the images, and the representations suffice to bridge between the modalities of SHG and BF. The best results for cross-modality retrieval of transformed full-sized images are obtained by our porposed method, using CoMIRs to learn representations for both input modalities, in combination with SURF to extract features for the BoW.

Similar behaviour to full sized image retrieval can be seen also in Table 1b, when querying patches. However for s-CBIR, ResNet as a dense feature extractor is not able to compete with SIFT and SURF. It extracts the same number of features regardless of the input image size. Hence, the resulting feature descriptor is of the same dimension for both the full sized images in the searchable repository, as well as for the query image. The average pooling layer of the network blurs out the features which result from the common region of the full sized image and the patch. This highlights the advantage of sparse feature extractors like SIFT and SURF.

Comparing the use of BoW models to a recently introduced RIS toolkit 2DKD (originally developed for within modality search), shows that the performance of 2DKD for cross-modal retrieval is higher using CoMIRs than using the original images, but the results are still significantly worse than the BoW based approaches. Even the within-modality retrieval performance of 2DKD is low. This is likely due to 2DKD extracting descriptors based on Krawtchouk polynomials, which can be seen as shape descriptors. The BF images used in this study are dense and their content corresponds rather to texture than shape, whereas SHG images are sparse and lack concrete shapes.

Furthermore, the proposed method outperforms the recent CBIR successfully used in medical image retrieval IMTDF, and the recent cross-modal image retrieval method TC-Net (Table 3). Similarly to the representation learning of CoMIRs used in our method, TC-NET uses a contrastive loss (triplet loss), but learns the feature embeddings used for retrieval directly. However, it uses a siamese network, i.e. all weights are shared for the networks streams processing the different modalities. We suspect that the weight sharing is the reason behind the lower retrieval success of TC-Net, as it can degrade performance when the modalities are very different in structure.

## Conclusion

We present a novel, data-independent approach for the challenging task of instance-level reverse image search across modalities without labels, and evaluate it on BF and SHG microscopy images used in histopathology. We combine the power of deep learning to generate representations for images of different modalities, with robust classical methods of feature extraction to create a BoW. Finally, we add re-ranking to further boost the retrieval success. Our proposed method outperforms two most recent approaches applicable to instance-level retrieval across BF and SHG modalities. Through a replacement study we confirm its efficacy and superiority over other design choices. We observe that using shape descriptors relying on Krawtchouk moments is inferior to BoW models for retrieval of BF and SHG images, and that in order to apply representation learning or I2I to bridge between modalities, it is essential that the learned representations are equivariant under the transformations between corresponding images. Furthermore, we show that it is crucial to use translation- and rotation-invariant feature extractors such as SIFT and SURF. Future work includes testing the pipeline on other modalities, and developing an improved feature selection and matching procedure tailored specifically for CoMIR type representations. In addition, the interplay between the raw modalities and their CoMIRs can be further explored in particular for modalities which share many features that may be lost or blurred in the current representation learning approach, to assess if a fused utilization can yield further improvements.

### Supplementary Information

Supplementary Information.

## Data Availability

The datasets used in the study are freely accessible in the zenodo repository, https://zenodo.org/record/3874362 and https://zenodo.org/record/5557568.

## References

[1] Müller, H., Michoux, N., Bandon, D. & Geissbuhler, A. A review of content-based image retrieval systems in medical applications-clinical benefits and future directions. *Int. J. Med. Inform.* **73**, 1–23 (2004). 15036075 10.1016/j.ijmedinf.2003.11.024

[2] Kapoor, R., Sharma, D. & Gulati, T. State of the art content based image retrieval techniques using deep learning: A survey. *Multimed. Tools Appl.* **80**, 29561–29583. 10.1007/s11042-021-11045-1 (2021). 10.1007/s11042-021-11045-1

[3] Qayyum, A., Anwar, S. M., Awais, M. & Majid, M. Medical image retrieval using deep convolutional neural network. *Neurocomputing* **266**, 8–20. 10.1016/j.neucom.2017.05.025 (2017). 10.1016/j.neucom.2017.05.025

[4] Mbilinyi, A. & Schuldt, H. Cross-modality medical image retrieval with deep features. In *International Conference on Bioinformatics and Biomedicine (BIBM)*, 2632–2639. 10.1109/BIBM49941.2020.9313211 (2020).

[5] Putzu, L., Loddo, A. & Ruberto, C. D. Invariant moments, textural and deep features for diagnostic MR and CT image retrieval. In *Computer Analysis of Images and Patterns*, 287–297 (Springer, 2021).

[6] Kong, B., Supancic, J. S., Ramanan, D. & Fowlkes, C. C. Cross-domain forensic shoeprint matching. In *British Machine Vision Conference (BMVC)* (2017).

[7] Sivic, J. & Zisserman, A. Efficient visual search of videos cast as text retrieval. *IEEE Trans. Pattern Anal. Mach. Intell.* **31**, 591–606 (2009). 19229077 10.1109/TPAMI.2008.111

[8] Philbin, J., Chum, O., Isard, M., Sivic, J. & Zisserman, A. Object retrieval with large vocabularies and fast spatial matching. In *2007 IEEE Conference on Computer Vision and Pattern Recognition*, 1–8 (2007).

[9] Caicedo, J. C., Cruz, A. & Gonzalez, F. A. Histopathology image classification using bag of features and kernel functions. In *Artificial Intelligence in Medicine*, 126–135 (Springer, 2009).

[10] Cao, B., Araujo, A. & Sim, J. Unifying deep local and global features for image search. In *Computer Vision—ECCV 2020* (eds Vedaldi, A. *et al.*) 726–743 (Springer, 2020).

[11] Hedge, N., Hipp, J. & Liu, Y. Similar image search for histology: SMILY. *npj Digit. Med.* **1**, 2. 10.1038/s41746-019-0131-z (2019). 10.1038/s41746-019-0131-z PMC6588631 31304402

[12] Komura, D. *et al.* Luigi: Large-scale histopathological image retrieval system using deep texture representations. *bioRxiv*10.1101/345785 (2018). 10.1101/345785

[13] Otálora, S., Schaer, R., Jimenez-del Toro, O., Atzori, M. & Müller, H. Deep learning based retrieval system for gigapixel histopathology cases and open access literature. *bioRxiv*10.1101/408237 (2018). 10.1101/408237 PMC6639847 31367471

[14] Chen, P. *et al.* Interactive thyroid whole slide image diagnostic system using deep representation. *Comput. Methods Programs Biomed.* **195**, 105630. 10.1016/j.cmpb.2020.105630 (2020). 32634647 10.1016/j.cmpb.2020.105630 PMC7492444

[15] Barisoni, L., Lafata, K. J., Hewitt, S. M., Madabhushi, A. & Balis, U. G. J. Digital pathology and computational image analysis in nephropathology. *Nat. Rev. Nephrol.* **16**, 669–685. 10.1038/s41581-020-0321-6 (2020). 32848206 10.1038/s41581-020-0321-6 PMC7447970

[16] Hristu, R. *et al.* Influence of hematoxylin and eosin staining on the quantitative analysis of second harmonic generation imaging of fixed tissue sections. *Biomed. Opt. Express* **12**, 5829–5843. 10.1364/BOE.428701 (2021). 34692218 10.1364/BOE.428701 PMC8515976

[17] Keikhosravi, A., Bredfeldt, J. S., Sagar, A. K. & Eliceiri, K. W. Chapter 28—Second-harmonic generation imaging of cancer. In *Quantitative Imaging in Cell Biology, Methods in Cell Biology*, Vol. 123, 531–546 (Academic Press, 2014). 10.1016/B978-0-12-420138-5.00028-8 24974046

[18] Zhou, X., Han, X., Li, H., Wang, J. & Liang, X. Cross-domain image retrieval: Methods and applications. *J. Multimed. Inf. Retr.* **11**, 199–218. 10.1007/s13735-022-00244-7 (2022). 10.1007/s13735-022-00244-7

[19] Liu, F. *et al.* Infrared and visible cross-modal image retrieval through shared features. *IEEE Trans. Circuits Syst. Video Technol.* **31**, 4485–4496. 10.1109/TCSVT.2020.3048945 (2021). 10.1109/TCSVT.2020.3048945

[20] Wu, A., Zheng, W.-S., Yu, H.-X., Gong, S. & Lai, J. RGB-infrared cross-modality person re-identification. In *International Conference on Computer Vision (ICCV)*, 5390–5399. 10.1109/ICCV.2017.575 (2017).

[21] Xiong, W., Xiong, Z., Cui, Y. & Lv, Y. A discriminative distillation network for cross-source remote sensing image retrieval. *IEEE J. Sel. Top. Appl. Earth Observ. Remote Sens.* **13**, 1234–1247. 10.1109/JSTARS.2020.2980870 (2020). 10.1109/JSTARS.2020.2980870

[22] Xiong, W., Lv, Y., Zhang, X. & Cui, Y. Learning to translate for cross-source remote sensing image retrieval. *IEEE Trans. Geosci. Remote Sens.* **58**, 4860–4874. 10.1109/TGRS.2020.2968096 (2020). 10.1109/TGRS.2020.2968096

[23] Li, Y., Zhang, Y., Huang, X. & Ma, J. Learning source-invariant deep hashing convolutional neural networks for cross-source remote sensing image retrieval. *IEEE Trans. Geosci. Remote Sens.* **56**, 6521–6536. 10.1109/TGRS.2018.2839705 (2018). 10.1109/TGRS.2018.2839705

[24] Lin, H. *et al.* TC-Net for ISBIR: Triplet classification network for instance-level sketch based image retrieval. In *Proceedings of ACM International Conference on Multimedia*, 1676–1684. 10.1145/3343031.3350900 (ACM, 2019).

[25] Zhang, J. *et al.* Generative domain-migration hashing for sketch-to-image retrieval. In *Computer Vision—ECCV 2018* (eds Ferrari, V. *et al.*) 304–321 (Springer, Cham, 2018).

[26] Bai, C., Chen, J., Ma, Q., Hao, P. & Chen, S. Cross-domain representation learning by domain-migration generative adversarial network for sketch based image retrieval. *J. Vis. Commun. Image Represent.* **71**, 102835. 10.1016/j.jvcir.2020.102835 (2020). 10.1016/j.jvcir.2020.102835

[27] Bui, T., Ribeiro, L., Ponti, M. & Collomosse, J. Compact descriptors for sketch-based image retrieval using a triplet loss convolutional neural network. *Comput. Vis. Image Underst.* **164**, 27–37 (2017). 10.1016/j.cviu.2017.06.007

[28] Lei, H. *et al.* A new algorithm for sketch-based fashion image retrieval based on cross-domain transformation. *Wirel. Commun. Mob. Comput.*10.1155/2021/5577735 (2021). 10.1155/2021/5577735

[29] Yang, E. *et al.* Deep Bayesian hashing with center prior for multi-modal neuroimage retrieval. *IEEE Trans. Med. Imaging* **40**, 503–513. 10.1109/TMI.2020.3030752 (2021). 33048672 10.1109/TMI.2020.3030752 PMC7909752

[30] Fang, J., Fu, H. & Liu, J. Deep triplet hashing network for case-based medical image retrieval. *Med. Image Anal.* **69**, 101981. 10.1016/j.media.2021.101981 (2021). 33588123 10.1016/j.media.2021.101981

[31] Pielawski, N. *et al.* CoMIR: Contrastive multimodal image representation for registration. In *Advances in Neural Information Processing Systems*, Vol. 33, 18433–18444 (Curran Associates, Inc., 2020).

[32] Bay, H., Ess, A., Tuytelaars, T. & Van Gool, L. SURF: Speeded up robust features. *Comput. Vis. Image Underst. (CVIU)* **110**, 346–359 (2008). 10.1016/j.cviu.2007.09.014

[33] Wetzer, E., Breznik, E., Lindblad, J. & Sladoje, N. Re-ranking strategies in cross-modality microscopy retrieval. In *IEEE ISBI 2022 International Symposium on Biomedical Imaging, 28–31 March, 2022, Kolkata, India* (Institute of Electrical and Electronics Engineers (IEEE), 2022).

[34] Eliceiri, K., Li, B. & Keikhosravi, A. Multimodal biomedical dataset for evaluating registration methods (patches from TMA cores). *zenodo* https://zenodo.org/record/3874362 (2020).

[35] Conklin, M. W. *et al.* Aligned collagen is a prognostic signature for survival in human breast carcinoma. *Am. J. Pathol.* **3**, 1221–1232. 10.1016/j.ajpath.2010.11.076 (2011). 10.1016/j.ajpath.2010.11.076 PMC3070581 21356373

[36] Jégou, S., Drozdzal, M., Vazquez, D., Romero, A. & Bengio, Y. The one hundred layers tiramisu: Fully convolutional densenets for semantic segmentation. In *Proceedings of CVPR Workshops*, 11–19 (2017).

[37] Isola, P., Zhu, J.-Y., Zhou, T. & Efros, A. A. Image-to-image translation with conditional adversarial networks. In *2017 IEEE Conference on Computer Vision and Pattern Recognition (CVPR)* (2017).

[38] Zhu, J.-Y., Park, T., Isola, P. & Efros, A. A. Unpaired image-to-image translation using cycle-consistent adversarial networks. In *International Conference on Computer Vision (ICCV)* (2017).

[39] Lowe, D. G. Object recognition from local scale-invariant features. In *Proceedings of International Conference on Computer Vision (ICCV)*, Vol. 2, 1150–1157 (1999).

[40] He, K., Zhang, X., Ren, S. & Sun, J. Deep residual learning for image recognition. In *2016 IEEE Conference on Computer Vision and Pattern Recognition (CVPR)*, 770–778. 10.1109/CVPR.2016.90 (2016).

[41] DeVille, J. S., Kihara, D. & Sit, A. 2DKD: A toolkit for content-based local image search. *Source Code Biol. Med.*10.1186/s13029-020-0077-1 (2020). 32064000 10.1186/s13029-020-0077-1 PMC7011505

[42] Babenko, A. & Lempitsky, V. Aggregating local deep features for image retireval. In *International Conference on Computer Vision (ICCV)* (2015).

[43] Bhandi, V. & Sumithra Devi, K. A. Image retrieval by fusion of features from pre-trained deep convolution neural networks. In *International Conference on Advanced Technologies in Intelligent Control, Environment, Computing Communication Engineering (ICATIECE)*, 35–40. 10.1109/ICATIECE45860.2019.9063814 (2019).

[44] Jun, H., Ko, B., Kim, Y., Kim, I. & Kim, J. Combination of multiple global descriptors for image retrieval. *CoRR* (2019).

[45] Sit, A. & Kihara, D. Comparison of image patches using local moment invariants. *IEEE Trans. Image Process.* **23**, 2369–2379. 10.1109/TIP.2014.2315923 (2014). 24718574 10.1109/TIP.2014.2315923

[46] Song, J., Yu, Q., Song, Y.-Z., Xiang, T. & Hospedales, T. M. Deep spatial-semantic attention for fine-grained sketch-based image retrieval. In *International Conference on Computer Vision (ICCV)*, 5552–5561. 10.1109/ICCV.2017.592 (2017).

