# Supplementary material for: Cross-modality sub-image retrieval using contrastive multimodal image representations
Source: Sci Rep. 2024 Aug 13;14:18798. doi: 10.1038/s41598-024-68800-1 (PMC11322435; doi:10.1038/s41598-024-68800-1)
Supplement: Supplementary file 1 — Supplementary Information. [file 41598_2024_68800_MOESM1_ESM.pdf]

A Appendix

A.1 CoMIR training

Contrastive Multimodal Image Representations (CoMIRs) were introduced by Pielawski & Wetzer et al.[1] and map two given images of different modalities to similar, abstract, 2D representations using contrastive learning for aligned multimodal image pairs. The contrastive loss is based on InfoNCE as described in Oord et al.[2] and is given by

$$\mathcal{L}_{\boldsymbol{\theta}}(\mathcal{D}) = -\frac{1}{n}\sum_{i=1}^{n}\left(\log \frac{e^{h(\mathbf{y}_i^1,\mathbf{y}_i^2)/\tau}}{e^{h(\mathbf{y}_i^1,\mathbf{y}_i^2)/\tau} + \sum_{\mathbf{y}_j^1,\mathbf{y}_j^2 \in \mathcal{D}_{neg}} e^{h(\mathbf{y}_j^1,\mathbf{y}_j^2)/\tau}}\right), \quad (1)$$

where $\mathcal{D} = \{(\boldsymbol{x}_i^1, \boldsymbol{x}_i^2)\}_{i=1}^n$ is an i.i.d. dataset containing n data points, $\boldsymbol{x}^j$ is an image in modality j, and $f_{\boldsymbol{\theta}_j}$ the network processing modality j with respective parameters $\boldsymbol{\theta}_j$ for $j \in \{1,2\}$. The exponential of a critic function $h(\mathbf{y}^1, \mathbf{y}^2)$ computes the similarity (here mean squared error) between CoMIRs $\mathbf{y}^1 = f_{\boldsymbol{\theta}_1}(\boldsymbol{x}^1)$ and $\mathbf{y}^2 = f_{\boldsymbol{\theta}_2}(\boldsymbol{x}^2)$ for the scaling parameter $\tau > 0$ (here $\tau = 0.5$ was used). As in other contrastive learning settings, representations are learnt which are similar for the so-called *anchor* and *positive*, and dissimilar for the *anchor* and *negative*, but that in the case of CoMIRs, the representations are 2D embeddings of the same spatial dimensions as the input images. A hyperpararmeter-free modification to the training regime was introduced to enforce rotational equivariance of CoMIRs by Pielawski & Wetzer et al[1]. The networks $f_{\boldsymbol{\theta}_1}$ and $f_{\boldsymbol{\theta}_2}$ processing the two modalities are fed differently rotated input per network (randomly chosen multiples of 90 degrees, independently per network/modality), and their features, i.e. CoMIRs at the end of the network are rotated back such that the representations are aligned, before applying the loss. This causes the networks to commute with rotations and thereby to generate rotationally equivariant representations. This approach was later extended to accomodate commutation by affine and deformable transformations by Nordling et al.[3].

A.2 Aerial Image Retrieval

A.2.1 Zurich Dataset

The aerial dataset consists of Near-Infrared (NIR) and three colour channels (in B-G-R order) images acquired by QuickBird of the city of Zurich. An example is shown in Fig. 1. It is released on Zenodo[4] as a multimodal registration benchmark, divided into 3 folds to allow for 3-fold cross validation. As the original images[5,6] vary in size, each image was subdivided into the maximal number of equal-sized non-overlapping regions such that each region can contain exactly one 300×300px image patch. Then one 300×300px image patch is extracted from the centre of each region. This splitting leads to each evaluation fold containing 72 test samples.

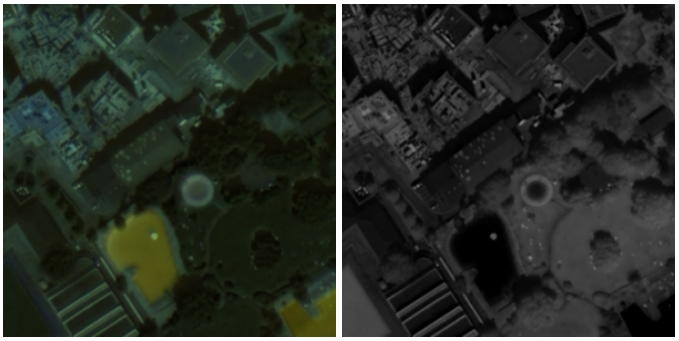

Figure 1. Example image pair of RGB (left) and NIR (right) images from the Zurich Dataset.

The dataset comes with different levels of transformations; the one denoted "tlevel 3" in the Zenodo repository was used in the experiments of this study. It was created by applying uniformly distributed rigid transformations to the images. Each image is randomly rotated by an angle $\theta \in [-20, 20]$ degrees (with bilinear interpolation), followed by translations in x and y directions by tx and ty pixels respectively, where tx and ty are randomly sampled within $[-28, 28]$.

A.2.2 Experiments

Our primary experiments on the biomedical dataset of SHG and BF modalities included both a replacement study and a comparison with two state-of-the-art methods. To demonstrate the generability of the proposed method, we apply it in the cross-modality retrieval task for a very different, non-biomedical application. As images from the Zurich dataset are quite small, sub-image retrieval was deemed unreasonable, and only full-image retrieval results are reported.

To show the applicability of our method and importance of the first stage of the pipeline – bridging the modality gap – even for rather similar modalities compared to the biomedical dataset, we report the results for the GAN representations also. We again compare our proposed pipeline (using CoMIRs) with using CycleGAN and Pix2Pix to learn the image representations, see Table 1 We also provide results of the competing, state-of-the-art methods, see Table 2.

As the dataset is relatively small and has been released in three folds, we run all experiments on all folds and results are reported per fold as well as averaged over all three folds ("mean folds"), and averaged over both directions, i.e. retrieving a query of modality A in modality B, and vice versa, as "mean overall".

Image Representations: The training details to create CoMIRs, CycleGAN and pix2pix of the Zurich images follow Lu et al.[7] using the same data augmentation for all three methods, which consists of random horizontal flip ($p = 0.5$); random rotations ($p = 1$, $\theta \in [-180°, 180°)$) using either ($p = 0.33$) nearest neighbour, linear, or cubic interpolation; random Gaussian blur ($p = 0.5$) with a standard deviation $\sigma \in [0, 2.0]$; and centre-crop ($p = 1$) to size of 256×256 px. Batch size for CoMIRs was 8, for CycleGAN 4 and pix2pix 64, in each case the largest batch size possible given the computational restrictions. The critic function to generate CoMIRs is based on mean squared error with $\tau = 0.5$ as identified most suitable in registration tasks[1,7]. Pix2pix and CycleGAN are trained for 200 epochs, in the case of CoMIRs patches are randomly sampled from the images during training for a total of 12800 iterations for CoMIRs as in Pielawski & Wetzer et al[7].

During inference the input images are padded to 512×512 px pixels using mirroring in case of pix2pix and CycleGAN, the padded areas are cropped again before performing the image retrieval task.

The results are shown in Table 1. We see that, despite the two modalities of the dataset being visually less challenging to match (see Fig. 1), the GAN-based representation learning does not perform satisfactorily. It further underlines the challenge of the generative task to not only find a representation that is in-distribution of the target modality, but to produce features that are locally corresponding to the features in the original image.

We tested the 2DKD toolbox on the Zurich dataset, but the performance w.r.t. Acc@1 was unsatisfactory and below random. One reason for the low performance could be attributed to the fact that Krawtchouk descriptors are considered shape descriptors and both the histological and the aerial dataset used in this study is defined by it dense texture rather than salient objects charazterized by their shape.

			Fold 1		Fold 2		Fold 3		Mean Folds	Mean Overall
			**Query**		**Query**		**Query**			
			RGB(T)	NIR(T)	RGB(T)	NIR(T)	RGB(T)	NIR(T)		
**Searchable Repository**	**CoMIR**	RGB	-	83.3	-	97.2	-	95.8	92.1	91.9
		NIR	84.7	-	94.4	-	95.8	-	91.6	
	**CycleGAN**	RGB	-	6.9	-	41.7	-	1.4	16.7	19.2
		NIR	15.3	-	43.1	-	6.9	-	21.8	
	**Pix2Pix**	RGB	-	44.4	-	37.5	-	33.3	38.4	38.6
		NIR	41.6	-	30.6	-	44.4	-	38.9	

Table 1. Image retrieval results on the Zurich dataset, using our proposed pipeline with either CoMIR representations (proposed) or GAN-based ones (alteratives). Only the mean Acc@1 (which equals mAP@1) for retrieval across modalities and transformations is reported.

Competing Methods: We again compare our pipeline to the TC-Net[8] and IMTDF[9]. The general settings for both methods are the same as in the main experiments (i.e. as in the original papers), with the exception of using a batch size of 8 and additionally lowering the weight for the centre loss by a factor of 10 in training the TC-Net (we have found these settings performed better).

Results in Table 2 once again show that our proposed pipeline outperforms the two competing methods by a large margin. On the other hand, all three methods perform much better compared to their performance on the biomedical dataset of BF and SHG images. This illustrates that the methods are appropriate for the cross-modal and cross-transformation retrieval when the two modalities show the same main structures. It also shows how challenging of a problem retrieval across the BF and SHG modalities really is.

			Fold 1		Fold 2		Fold 3		Mean Folds	Mean Overall
			**Query**		**Query**		**Query**			
			RGB(T)	NIR(T)	RGB(T)	NIR(T)	RGB(T)	NIR(T)		
**Searchable Repository**	**Ours**	RGB	-	83.3	-	97.2	-	95.8	92.1	91.9
		NIR	84.7	-	94.4	-	95.8	-	91.6	
	**TC-Net**	RGB	-	80.6	-	79.2	-	94.4	84.7	82.7
		NIR	66.7	-	80.6	-	94.4	-	80.6	
	**IMTDF**	RGB	-	63.9	-	54.2	-	51.4	56.5	44.7
		NIR	26.4	-	45.8	-	26.4	-	32.9	

Table 2. Image retrieval results on the Zurich dataset, using the competing methods of TC-Net and IMTDF. Results using our proposed method are reported again, for easier comparison. Only the mean Acc@1 (which equals mAP@1) for retrieval across modalities and transformations is reported.

References

1. Pielawski, N. *et al.* CoMIR: Contrastive multimodal image representation for registration. In *Advances in Neural Information Processing Systems*, vol. 33, 18433–18444 (Curran Associates, Inc., 2020).
2. Oord, A. v. d., Li, Y. & Vinyals, O. Representation learning with contrastive predictive coding. *arXiv preprint arXiv:1807.03748* (2018).
3. Nordling, L., Öfverstedt, J., Lindblad, J. & Sladoje, N. Contrastive learning of equivariant image representations for multimodal deformable registration. In *2023 IEEE 20th International Symposium on Biomedical Imaging (ISBI)*, 1–5 (IEEE, 2023).
4. Lu, J., Öfverstedt, J., Lindblad, J. & Sladoje, N. Datasets for Evaluation of Multimodal Image Registration, DOI: 10.5281/zenodo.5557568 (2021).
5. Michele Volpi, V. F. Zurich summer dataset, DOI: 10.5281/zenodo.5914759 (2022).
6. Volpi, M. & Ferrari, V. Semantic segmentation of urban scenes by learning local class interactions. In *Proceedings of the IEEE Conference on Computer Vision and Pattern Recognition Workshops*, 1–9 (2015).
7. Lu, J., Öfverstedt, J., Lindblad, J. & Sladoje, N. Is image-to-image translation the panacea for multimodal image registration? a comparative study. *Plos one* **17**, e0276196 (2022).
8. Lin, H. *et al.* TC-Net for ISBIR: Triplet classification network for instance-level sketch based image retrieval. In *Proc. ACM Intl. Conf. on Multimedia*, 1676–1684, DOI: 10.1145/3343031.3350900 (ACM, 2019).
9. Putzu, L., Loddo, A. & Ruberto, C. D. Invariant moments, textural and deep features for diagnostic MR and CT image retrieval. In *Computer Analysis of Images and Patterns*, 287–297 (Springer Intl. Publishing, 2021).
